# Steroid Hormones as Modulators of Emotional Regulation in Male Urogenital Cancers

**DOI:** 10.1007/s12529-022-10139-w

**Published:** 2022-12-02

**Authors:** Pinuccia Faviana, Laura Boldrini, Lisa Gronchi, Luca Galli, Paola Erba, Carlo Gentile, Piero Vincenzo Lippolis, Elio Marchetti, Iosè Di Stefano, Enrico Sammarco, Alex D. Chapman, Massimo Bardi

**Affiliations:** 1 Department of Surgical, Medical, Molecular Pathology and Critical Area, University of Pisa, Via Roma, 57, Pisa, Italy; 2 Department of Translational Research and New Technologies in Medicine and Surgery, University of Pisa, Via Roma, 57, Pisa, Italy; 3 Istituto Europeo Di Oncologia, Via Ripamonti 435, I-20132 Milan, Italy; 4 General and Peritoneal Surgery, Department of Surgery, AOUP, Via Paradisa 2, Pisa, Italy; 5 Department of Psychology and Neuroscience, Randolph-Macon College, Ashland, VA 23005 USA

**Keywords:** Resiliency, DHEA, Cortisol, Urogenital cancer

## Abstract

**Background:**

Tumors develop within an organism operating in a specific social and physical environment. Cortisol and dehydroepiandrosterone (DHEA), two of the most abundant steroid hormones in humans, are involved in both emotional regulation and the tumor progression. Several studies reported preclinical findings that DHEA can have preventive and therapeutic efficacy in treating major age-associated diseases, including cancer, although the mechanisms of action are not yet defined. The main aim of current study was to investigate the relationship between psychological and physiological emotional regulation and cancer development.

**Method:**

This study assessed the quality of life of urogenital cancer male patients using several validated tools, including the Functional Assessment of Cancer Therapy-General and the Profile of Mood States. Saliva samples were collected to monitor peripheral activity of both cortisol and DHEA. It was hypothesized that patients with a better quality of life would have higher levels of the DHEA/cortisol ratios.

**Results:**

We found that the quality of life was positively related to DHEA, but not cortisol levels. Negative mood increases were related to lower levels of DHEA. Logistic regression of the predictors of metastases indicated three main independent factors involved: DHEA, age, and cortisol. In other words, the higher the DHEA levels in comparison to cortisol levels, controlling for age, the lower the probability of metastases.

**Conclusion:**

Our results appear to support the hypothesis that emotional dysregulation mediated by DHEA/cortisol activity is a key factor in the probability of metastasis in urogenital cancers.

## Introduction

There is growing evidence that psychosocial interventions aimed to increase emotional regulation can have measurable benefits for people affected by cancer, including improved symptoms of mental health and well-being, optimized immune responses, and an overall better prospective for recovery [1, 2]. Early studies identified a small but significant connection between psychosocial disorders, such as depression, chronic stress, and social isolation, and various types of cancer [3–5]. It has been clear for quite sometimes that the link between psychosocial factors and increasing cancer risks could be mediated by the cellular microenvironment at the tumor level [6, 7]. Tumors develop within an organism living and operating within a social and physical environment [8]; thus, a more “bioecological” perspective, linking significant life events, personality and emotional regulation, and the quality of life during cancer treatment could be very useful in assessing cancer development and survivorship [9, 10]. These studies brought a conceptual shift on how to study neoplastic tissues, focusing not only on the cancer cells, but also on the cellular context in which tumors thrive [11] as well as the life events that can directly influence the allostatic loads of organisms [12]. It is still unclear if the well-known connection between emotional regulation and immune-endocrine functions [13, 14] can explain how psychosocial disorders can influence cancer progression of urogenital cancers (UCs). These tumors account for approximately 14% of all human cancers in industrialized countries [15]. Kidney, prostate, testicular, and bladder tumors are the most common UC types and are among the 10 most prevalent cancers in men. They represent a heterogeneous set of diseases with different prognosis and therapeutic approaches, and thus an ideal candidate to investigate their relationship with resiliency, defined as the psychological and physical ability of an organism to cope in the face of significant challenges and still being able to thrive [16].

Considering the multifaceted responses of coping mechanisms, it is important to reconcile both psychological and biological aspects of resiliency. From a biological perspective, when an organism is challenged with a stressful event, the psychological and behavioral response strategies determine its ability to successfully cope with such event. When these responses are appropriately tailored to the anticipated needs, the organism can achieve strategic psychological and physiological modifications to maintain healthy outcomes [17–19]. Alternatively, negative health outcomes can emerge when physiological demands are not sufficiently anticipated, resulting in dysregulated cellular microenvironment that could ultimately increase tumor proliferation [20]. Finding reliable and theoretical valid operational definitions of both psychological and physiological resilience is therefore imperative to assess the relationship with various types of cancer. In the current study, we selected several validated tools to assess the role of life events, personality traits, emotional responses, and the quality of life after being diagnosed with cancer. Taken together, these measures can represent a reliable index of emotional regulation.

To monitor emotional regulation from a physiological perspective, biomarkers of the hypothalamic–pituitary–adrenal (HPA) axis are often used [21]. Cortisol (CORT) and dehydroepiandrosterone (DHEA), including its sulfate form (DHEA-S), are two of the most abundant steroid hormones in human plasma and are both released in response to challenging events and an effective marker of disturbance in homeostatic balance [22]. Although both DHEA and DHEA-S are secreted by the adrenal gland, the sulfate form is about 250 times more concentrated and mostly independent by diurnal cycles; DHEA-S can be metabolized back to DHEA by sulfohydrolases in peripheral adrenal tissue [22]. Considering that CORT and DHEA are associated with different aspects of the stress response, they can be assessed in parallel to differentiate between the positive and negative effects of the HPA axis activation [23]. Abnormal levels of CORT can be a sign of cancer progression and prognosis [24]. For instance, differences in expression of CORT receptors in malignant and non-malignant tissue can be used to assess cancer prediction, diagnosis, and management [25]. DHEA is released during a stress response to inhibit both catecholamine upregulation in the adrenal medulla, as well as many of the negative effects of glucocorticoids in various tissues, including inflammation dysregulation in the TME [22, 26, 27]. Potentially related to the reduction in stress response and anxiety-like behaviors, research has demonstrated that DHEA can act centrally to decrease glucocorticoid-induced neuronal death in various brain regions associated with emotional and cognitive regulation, in addition to promoting neurogenesis and physiological resiliency [28, 29]. Indeed, the ratio between DHEA and CORT has been found to be a reliable index of neuroprotection [30, 31], and due to their general physiological effects, also related to cancer progression [32, 33]. To better cope with a continuously changing physical and social environment, humans have developed a dynamic interaction between the different cell types that comprise the immune system and other key neuroendocrine regulatory systems, such as the HPA axis. Compelling evidence has been found suggesting that the cells of the immune system operate similarly to a sensory organ informing the brain of inflammatory conditions [34]. Considering that recent evidence strongly supports the opinion that immune system has both positive and negative effects on tumorigenesis, and the inflammatory microenvironment is an essential component for tumors [35], it is imperative to better understand the dynamic communication between various forms of emotional regulation and cancer development.

The main aim of current study was to investigate the relationship between psychological and physiological emotional regulation and cancer development. Male patients affected by various types of UC were assessed during a follow-up clinical visit. It was hypothesized that the ability of people to cope with the imbalance provoked by both stress and the disease, as both physiological homeostasis and emotional regulation, would be related to each other. In other words, we predicted that patients with an overall better perception of their condition and quality of life, as measured by validated self-report questionnaires, would also have higher levels of the DHEA in comparison to CORT levels. Vice versa, patients with higher levels of DHEA/CORT ratios would have also better coping skills. Due to the correlational nature of the present study, we cannot test the directionality of this relationship, although based on previous studies [28–31], we believe that physiological activity of the HPA axis and emotional regulation influence each other. It was also hypothesized that patients with higher coping skills and higher DHEA/CORT ratios would also have a better chance to recover, as measured by the presence or absence of metastases.

## Methods

### Ethical Statement

This study was approved by the University of Pisa Medical School Ethical Committee (ID # 19005), and it has therefore been performed in accordance with the principles embodied in the World Medical Association Declaration of Helsinki. All participants gave their informed consent prior to their inclusion in the study.

### Participants and Procedure

Participants were recruited among patients with four types of urogenital cancer: testicular cancer (TC, *n* = 15), prostate cancer (PC, *n* = 69), renal cancer (RC, *n* = 34), and bladder cancer (BC, *n* = 19) treated at the Departments of Surgical, Medical, Molecular Pathology and Critical Area and Department of Translational Research and Advanced Technologies in Medicine, University of Pisa. Patients were considered eligible to participate if they did not use medications that could interfere with the HPA axis activity. In total, one hundred thirty-seven patients were recruited (mean age = 66.9 ± 14.9 SD years) as cancer patients and another 21 volunteers matched in age and not affected by cancer as a comparative group (mean age = 67.3 ± 8.8 SD years). The assessment for this study was completed within 2 years from the diagnosis (range: 3 to 24 months – mean time = 5.82 ± 4.8 SD months). Age was the only demographic factors included in the study.

Participants were tested on a week-day morning between 9:00 am and 12:00 pm to minimize circadian variations of steroid levels. Because the collection time was at least 2 h after awakening, cortisol awakening response was not an issue. On arrival, participants initially rested for a few minutes while they were informed of the procedure and of the general goals of the research. After they gave their informed consent, the questionnaires were administered. After that, saliva samples were collected by passive drooling. Participants were given the instructions to hold a short straw in their mouth with the test tube at the end and let the saliva drool into the test tube. The average completion time was less than 30 min per patient, and the whole procedure lasted between 30 and 45 min. Samples were stored at −70 °C within 30 min of their collection.

### Self-report Assessments

The Functional Assessment of Cancer Therapy-General (FACT-G) was designed to measure four domains of quality of life in cancer patients: physical, social, emotional, and functional well-being [36]. The FACT-G comprises four subscales: physical well-being, social/family well-being, emotional well-being, and functional well-being. From the data, we were able to generate an overall score with range and distribution specific to our sample.

The Profile of Mood States (POMS) [37] and the Personality Belief Questionnaire–Short Form (PBQ–SF) [38] were developed as clinical and research instruments to assess dysfunctional emotional regulation and beliefs associated with individual personality disorders. In this assessment of trait dysfunctional beliefs, each disorder was linked to specific behavioral markers corresponding to dysfunctional assumptions. For instance, the behavioral manifestations of dependent personality disorder related to submissiveness and excessive reliance on the approval and support of others were manifested with beliefs such as “I’m helpless and can’t cope as other people can.” As another example, behavioral correlates of narcissistic personality disorder were associated with underlying beliefs such as “Because I am special, others should put my wants above theirs.” When used in cancer research, these assessments were mostly used in relation to the ability of patients to deal with negative biopsy results [39]. The POMS questionnaire was employed to assess current mood states and mood changes in our sample [40]. These scales have been found to be consistent and reliable in both clinical and non-clinical samples, including patients with cancer [41] and immune dysfunctions [42]. The PBQ-SF questionnaire was used to assess potential sets of dysfunctional beliefs associated with cancer in our sample [43].

The Life Events Checklist for *DSM-5* (LEC-5) is a comprehensive screening instrument used to detect exposure to a range of potentially traumatic, life changing events [44, 45]. Despite its widespread use in studies focused on posttraumatic stress symptoms and disorders [46], this instrument has not been used as much to assess psychosocial distress among oncology patients, even though a large proportion of people diagnosed with cancer experience levels of distress that would benefit from psychosocial interventions [47], and a number of large-scale epidemiological studies have revealed that potentially traumatic event exposure is unfortunately quite prevalent, with over 60% of the population having experienced a significant traumatic event in their lifetime [44]. In our study, we used this checklist as a 16-item questionnaire.

### Endocrinological Assessments

Prior to the assays, saliva samples were shaken vigorously in a mixer for approximately 30 s. Next, the tube was centrifuged for 15 min at 1250* g*. Using a transfer pipette, the supernatant was transferred to a 13 × 100 mm glass test tube. This process was repeated three times to assure no contaminations were introduced in the final steps. The final steps of the assay procedure were performed according to the instructions of the manufacturer of the assay kit (Salimetrics, State College, PA, USA). Sample readings were completed using an automated micro-plate reader (BioTek Instruments, Winooski, VT, USA; model: Hybrid), and the Gen5 software (BioTek, BioTek Instruments, Winooski, VT, USA; version 2.01). Readings were assessed at a wavelength of 450 nm, with secondary filter corrections at 490 to 492 nm.

To assess the reliability of the saliva samples, we ran multiple quality controls [48] and then the Cronbach’s coefficient α was calculated. Quality controls were determined using 20 replicates at five different concentrations for each hormone. Intra-assay precision showed an average coefficient of variation of 3.9% for DHEA, 4.2% for DHEA-S, and 3.5% for CORT. The inter-assay precision was calculated in a similar way and returned the following percentages: 4.5% for DHEA, 6.1% for DHEA-S, and 3.8% for CORT. Cronbach’s α was > 0.80 for all tests. Recovery rate was calculated to assess the ability of the test to measure accurately the target hormone from saliva. To calculate recovery rate, five samples containing different levels of an endogenous hormone were spiked with known quantities of the same hormone and assayed. Average recovery ranges from 95 to 110% for all three hormones, thus demonstrating that the assays were accurate. The functional sensitivity of the kits was as follows: 8.32 pg/mL for DHEA, 198.3 pg/mL for DHEA-S, and 0.018 µg/dL for CORT.

### Statistical Analysis

Analysis of variance and *t*-test were used to analyze the difference in the averages by cancer type and presence or absence of metastases. Correlation among variables was assessed using Pearson’s product-moment coefficients. Analysis of covariance was used to control for the age of the participants. Although using many different tests to extract information from the same database can increase the probability to type II errors, we used these many analyses as general guidance for the multivariate test explained below. Nevertheless, significance levels between 0.05 and 0.01 should be taken cautiously. Missing values (below 2% of the data) were excluded by the analyses. All tests were two-tailed, and the significance threshold was set at α = 0.05.

To evaluate the internal consistency of all self-reported measures, we calculated the Cronbach’s α. This measure is computed by correlating the score for each item with the total score for each observation, and then comparing it the variance for all individual scores. The resulting coefficient ranges from 0 (all the items are independent from each other) to 1 (all the items have high covariance). Generally, coefficients higher than 0.60 are considered acceptable and higher than 0.80 are considered representing high internal consistency. All four self-reported measures in our dataset returned acceptable internal consistency, ranging from 0.67 (LEC-5) to 0.89 (FACT-G).

Logistic regression analysis was used to identify the best predictors of the binary dependent variable presence (1) or absence (0) of metastases. We selected this approach to provide a multivariate model able to summarize the many univariate analyses presented previously, thus reducing the probability of an inflated significance estimation. Predictors were DHEA, DHEA-S, CORT, age, and the self-report measures. The Wald statistic was used to assess the significance of each coefficient. The overall goodness-of-fit was assessed by the −2 Log Likelihood and by the Hosmer and Lemeshow test. The percentage of variance explained by each model was assessed using the Cox and Snell *R*^2^ and the Nagelkerke *R*^2^. The predictive accuracy of the models was determined by the hit ratio that is the percentage of cases correctly classified.

All statistical analyses were conducted using the SPSS computer program (IBM, Chicago, IL, version 27.0).

## Results

### Psychological Assessments

Cancer patients reported a significantly lower scores in the FACT-G questionnaire than controls (mean scores ± SD: 60.8 ± 10.3 vs. 76.8 ± 10.4; *t*_155_ = 42.8, *p* < 0.001). Although the number of traumatic events was higher in cancer patients, the overall total difference reported in the LEC-5 questionnaire was not significant (mean number of events ± SD: 3.28 ± 1.9 vs. 2.81 ± 1.7; *t*_155_ = 1.08, *p* = 0.301). No significant differences in both mood states and personality beliefs were found (POMS: 46.9 ± 15.4 vs. 52.7 ± 20.9; *t*_155_ = 2.24, *p* = 0.136; PBQ-SF: 17.9 ± 10.6 vs. 18.8 ± 10.2; *t*_155_ = 0.19, *p* = 0.662).

**Fig. 1:**
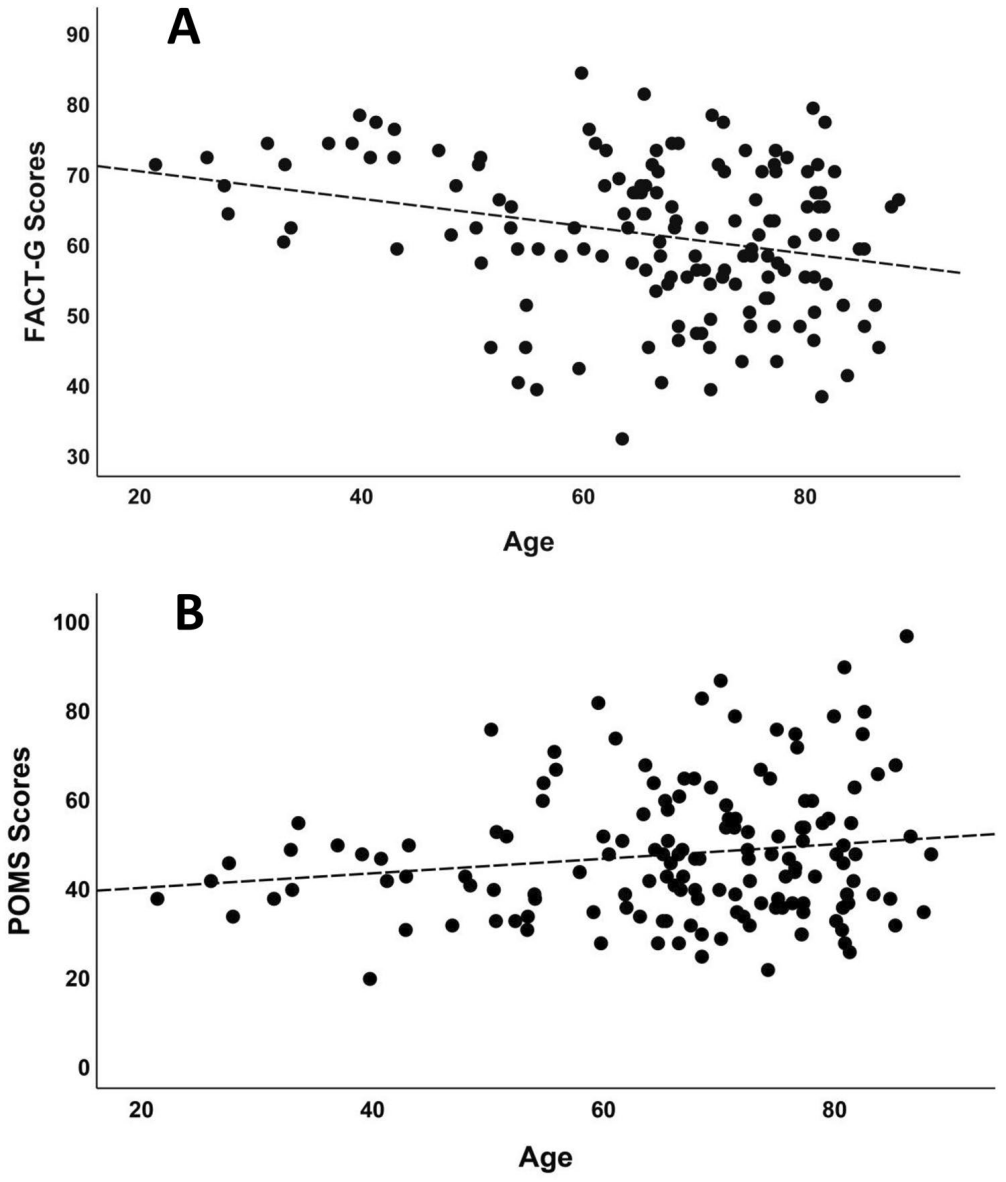
Correlation between age (in years) and self-reported measures (scores). **A** An inverse correlation between age and quality of life (FACT-G) was found (*r* = -0.267, *p* = 0.001). **B** A positive correlation between age and mood changes (POMS) was also found (*r* = 0.187, *p* = 0.05)

**Fig. 2:**
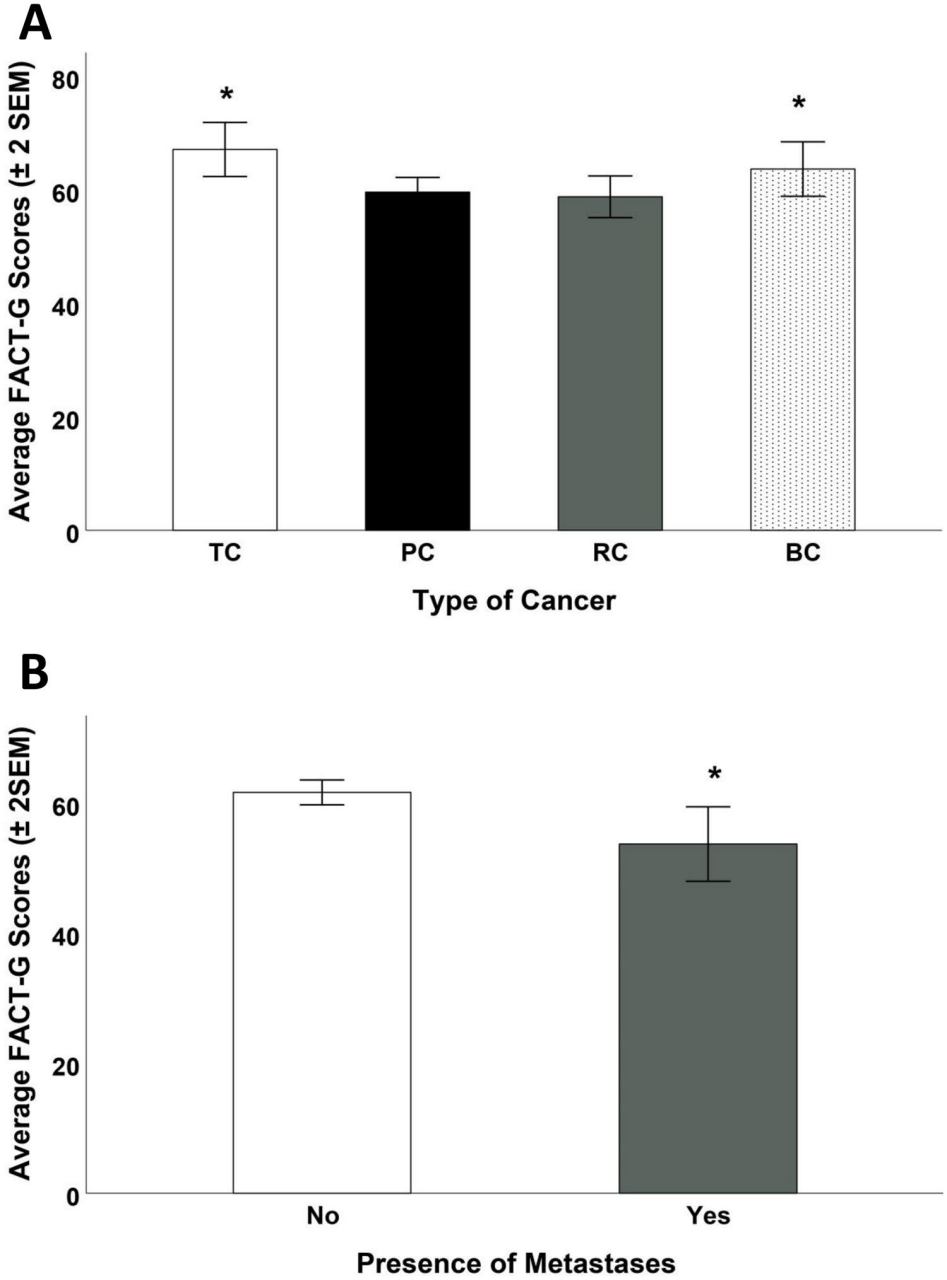
(**A**) Relationship between quality of life (FACT-G scores) and cancer type (TC = Testicular Cancer; PC = Prostate Cancer; RC = Renal Cancer; BC = Bladder Cancer). (**B**) Relationship between quality of life (FACT-G scores) and presence of metastases. (*) = *p* < 0.05

Focusing on cancer patients, it was found that age was inversely correlated with FACT-G (*r* = − 0.278, *p* < 0.001—Fig. 1A) and positively correlated with mood states (*r* = 0.175, *p* = 0.046—Fig. 1B) but it was not related to the other measures (LEC-5: *p* = 0.576; PBQ-SF: *p* = 0.377). Both significant relationships indicated a clear heteroscedasticity in the distribution, showing that the quality of life and mood states greatly increased variability as patients became older. The number of years from the diagnosis were not related to any of the four self-assessment measures (all *p*-values > 0.304). The type of cancer influenced the quality of life of the male patients as measured by the FACT-G questionnaire (*F*_3,133_ = 3.07, *p* = 0.030); post hoc test indicated that patients affected by TC and BC reported a higher quality of life than those affected by PC and RC (Fig. 2A). The presence of one or more metastases was also significantly related to the quality of life (*t*_135_ = 3.86, *p* = 0.033—Fig. 2B), but not to the other three self-assessment measures (all *p*-values > 0.247).

### Physiological Assessments

A significant correlation between DHEA and DHEA-S was found (*r* = 0.708, *p* < 0.001), whereas CORT was not significantly related to either measure (*p* > 0.373). Both DHEA and DHEA-S decreased significantly with age (*r* = −0.529, *p* < 0.001 and *r* = −0.336, *p* < 0.001 respectively), but not such a decline was found for CORT (*p* = 0.815). The number of years passed since the first diagnosis was inversely related to DHEA levels (*r* = −0.171, *p* = 0.048) but not to DHEA-S (*p* = 0.167) nor CORT (*p* = 0.168) levels.

Type of cancer was related to both DHEA and DHEA-S levels (*F*_3,131_ = 10.02, *p* < 0.001; and *F*_3,131_ = 2.8, *p* = 0.048), but not to CORT levels (*F*_3,131_ = 2.13, *p* = 0.099), although it could be noted a statistical trend toward significance. Bonferroni post hoc tests revealed that TC patients had the highest levels of both DHEA and DHEA-S. Since the highest values were recorded in TC patients, who were also significantly younger on average, analyses of covariance were run to control for the effect of age on these relationships. Results indicated that when controlling for age, both DHEA and DHEA-S relationships were no longer significant (*p* > 0.642).

**Fig. 3:**
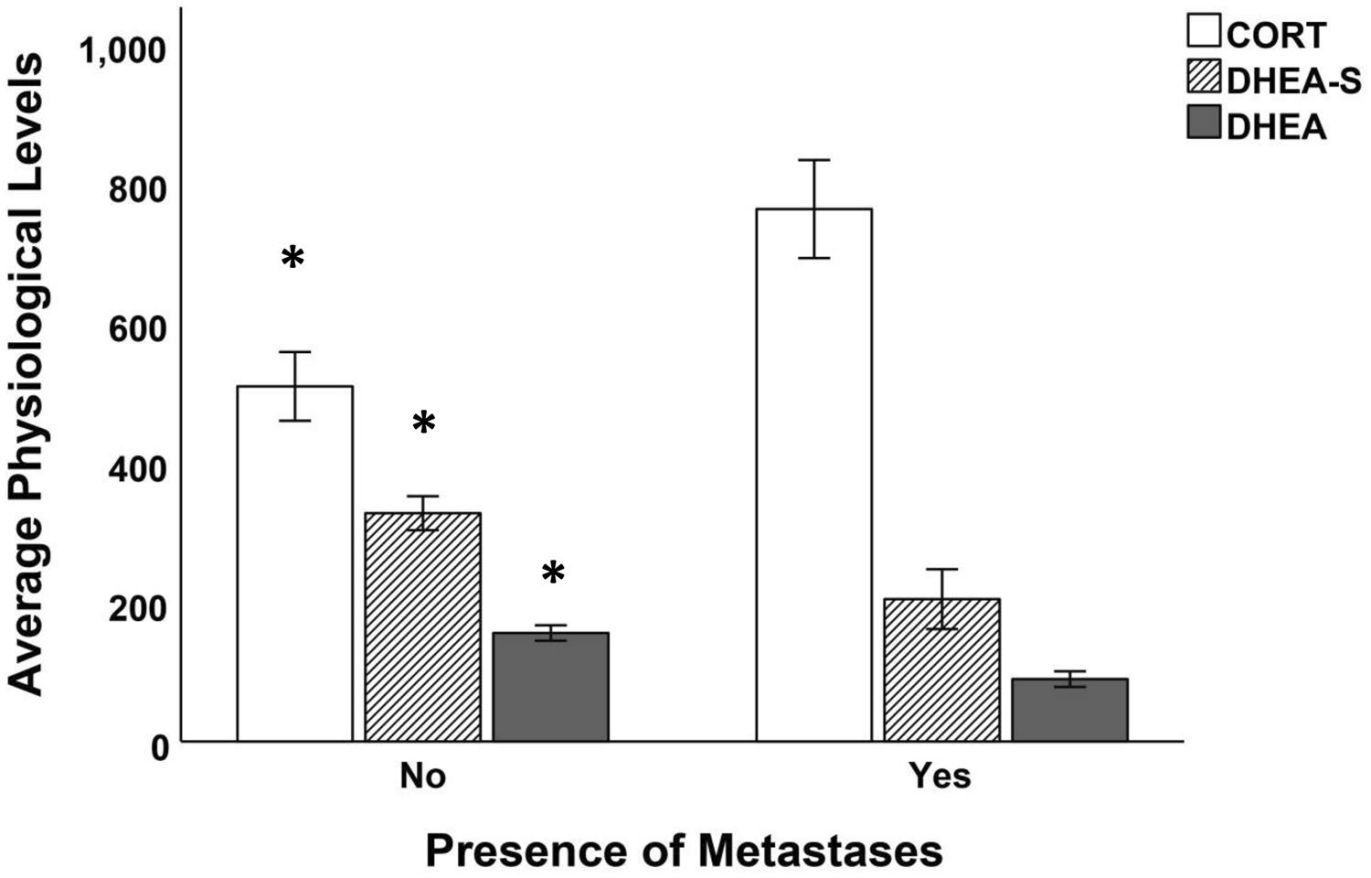
Physiological levels by presence of metastases. CORT was significantly higher on patients with metastases (*t*_130_ = 22.09, *p* < 0.001), whereas both DHEA and DHEA-S were significantly lower (*t*_130_ = 31.07, *p* < 0.001; DHEA-S: *t*_130_ = 20.28, *p* < 0.001)

All physiological markers were significantly associated with metastases (DHEA: *t*_133_ = 18.2, *p* < 0.001; DHEA-S: *t*_133_ = 15.1, *p* < 0.001; CORT: *t*_133_ = 14.2, *p* < 0.001). CORT was significantly higher on patients with metastases, whereas both DHEA and DHEA-S were significantly lower (Fig. 3).

### Psychological and Physiological Correlations

**Fig. 4:**
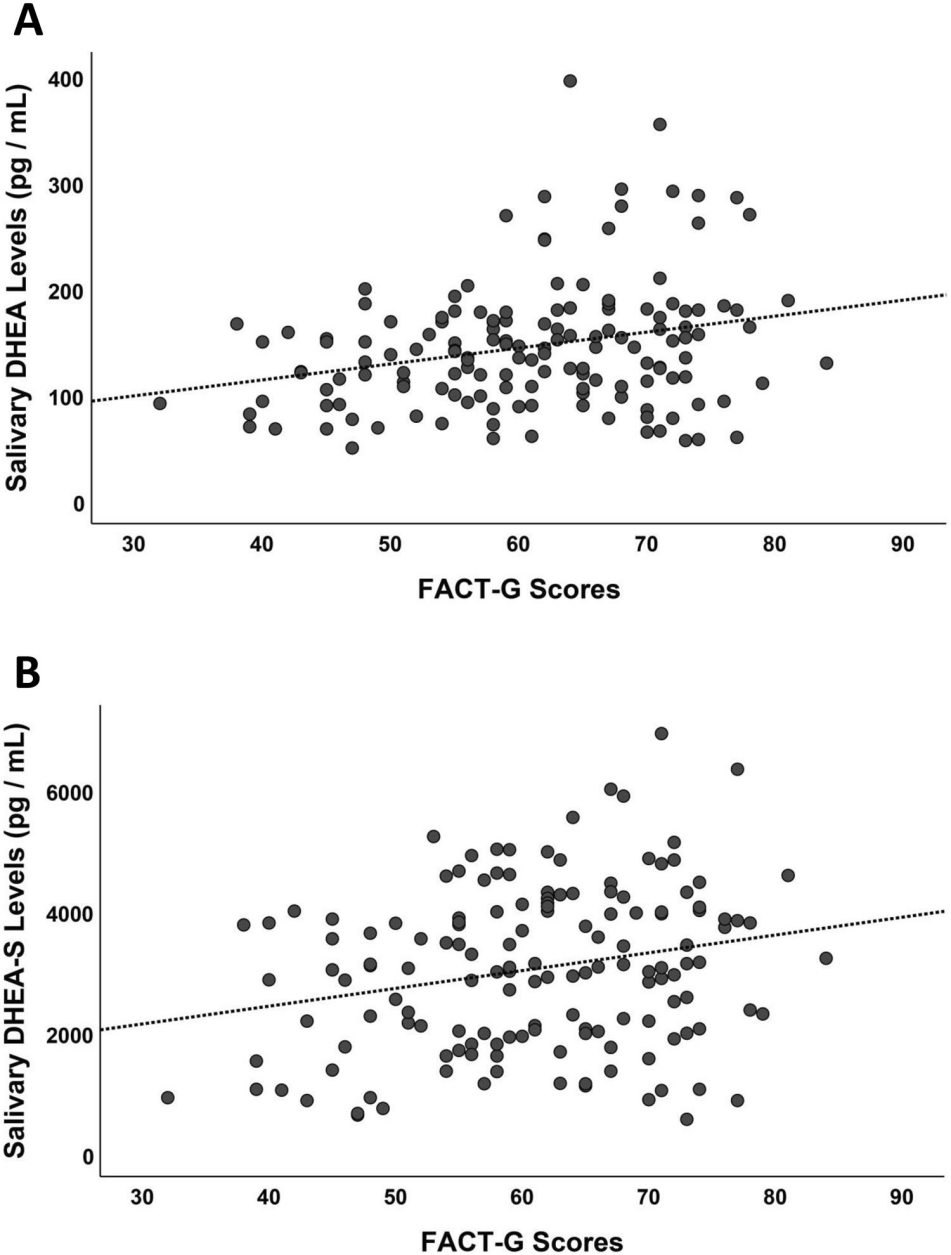
Correlation between DHEA/DHEA-S and quality of life (FACT-G scores). There was a positive correlation with both DHEA (*r* = 0.263, *p* = 0.001) (**A**) and DHEA-S (*r* = 0.233, *p* = 0.004) (**B**)

**Fig. 5:**
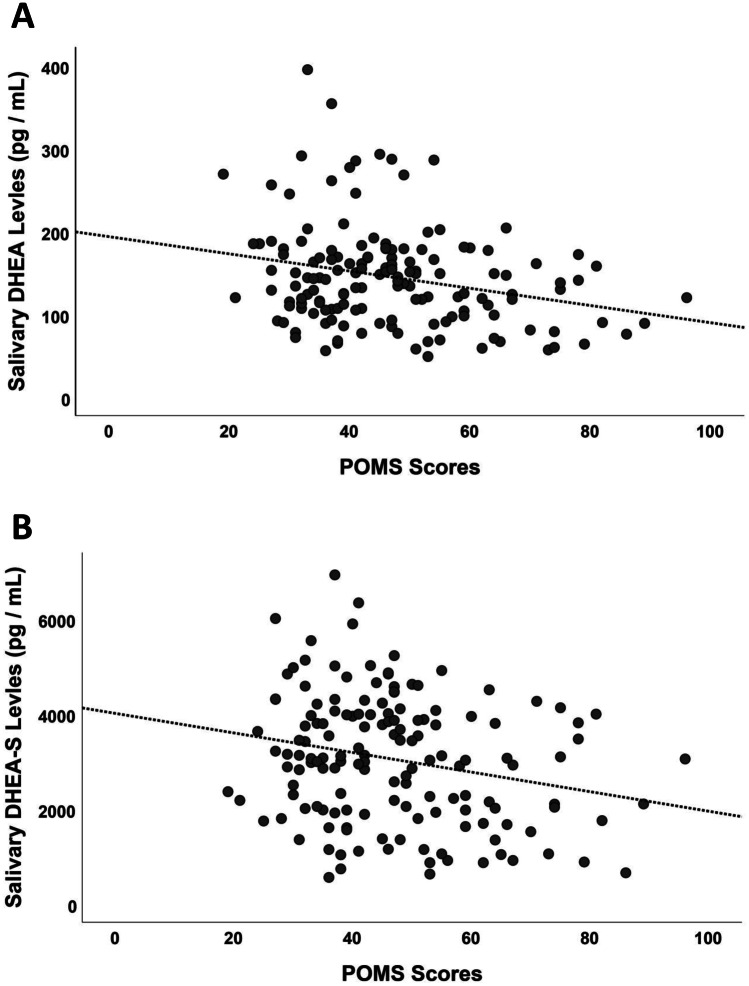
Correlation between DHEA / DHEA-S and mood changes (POMS scores). Negative correlations between mood states and DHEA ((*r* = − 0.259, *p* = 0.001) (**A**) and DHEA-S (*r* = − 0.243, *p* = 0.003) (**B**)

The quality of life reported using the FACT-G scores was positively correlated with both DHEA and DHEA-S, but not with CORT levels (*r* = 0.299, *p* < 0.001; *r* = 0.235, *p* = 0.006; *r* = −0.050, *p* = 0.562; Fig. 4). Negative correlations between mood states (POMS questionnaire) and DHEA and DHEA-S were also found (*r* = −0.302, *p* < 0.001; *r* = −0.258, *p* = 0.003—Fig. 5). Once again, CORT was not related to the self-assessment of mood state in our sample (*r* = −0.071, *p* = 0.425). The other two self-assessment measures were not significantly related to physiological values (all *p*-values > 0.121).

### Predictors of Metastases

Logistic regression was used to identify the best predictors of metastases in our sample. A stepwise forward method was used to compare several alternative models. The predictors selected were age, physiological levels, and the FACT-G and POMS scores since they were significantly related to each other. Three significant models were found (Table 1). In step 1, only DHEA was included. Goodness-of-fit tests indicated that this was a significant relationship (−2 Log likelihood = 86.19, *p* < 0.001; Hosmer and Lemeshow *χ*_8_^2^ = 5.94, *p* = 0.766). The percentage of variance explained was about 30% (Cox and Snell *R*^2^ = 0.276; Nagelkerke *R*^2^ = 0.316). This model was able to classify correctly 88.1% of cases. In step 2, the variable age was included and both the goodness-of-fit (−2 Log likelihood = 64.13; Hosmer and Lemeshow *χ*_8_^2^ = 5.34, *p* = 0.712) and the percentage of variance explained increased (Cox and Snell *R*^2^ = 0.325; Nagelkerke *R*^2^ = 0.512). Predictive accuracy of this model reached 90.9%. In the final step, CORT was included as well, increasing once more the goodness-of-fit (−2 Log likelihood = 45.15; Hosmer and Lemeshow *χ*_8_^2^ = 4.22, *p* = 0.85) and the variance explained (Cox and Snell *R*^2^ = 0.488; Nagelkerke *R*^2^ = 0.722). The overall percentage of correct cases increased to 94.8%. The other variables (DHEA-S, FACT-G, and POMS) were all excluded from the models.

**Table 1:** Stepwise logistic regression

Variables in the Equation									
		*B*	S.E	Wald	df	Sig	Exp(B)	95% C.I. for EXP(B)	
								Lower	Upper
Step 1^a^	DHEA	−0.049	0.010	22.248	1	0.000	0.952	0.933	0.972
	Constant	4.173	1.097	14.477	1	0.000	64.911		
Step 2^b^	DHEA	− 0.075	0.017	19.980	1	0.000	0.928	0.898	0.959
	Age	−0.120	0.034	12.201	1	0.000	0.887	0.829	0.949
	Constant	14.985	3.646	16.893	1	0.000			
Step 3^c^	DHEA	−0.074	0.019	15.692	1	0.000	0.929	0.895	0.963
	CORT	0.005	0.002	8.990	1	0.003	1.005	1.002	1.009
	Age	−0.134	0.038	12.164	1	0.000	0.875	0.812	0.943
	Constant	12.323	3.866	10.161	1	0.001			

^a^Variable(s) entered on step 1: DHEA

^b^Variable(s) entered on step 2: ETA

^c^Variable(s) entered on step 3: CORT

## Discussion

In the current study, we investigated the relationship between psychological and physiological emotional regulation in UC male patients. It was found that the quality of life of the patients, as measured by the FACT-G questionnaire, was positively correlated to both DHEA and DHEA-S, but not CORT levels at the time of the follow-up visit. Negative mood increases in the patients (POMS scores) were correlated to lower levels of DHEA and DHEA-S as well. Logistic regression of the predictors of metastases indicated three main independent factors involved: DHEA, age, and CORT. In other words, the higher the DHEA levels in comparison to CORT levels, controlling for age, the lower the probability of metastases in our small sample of males. Interestingly, subjective experiences (FACT-G and POMS) were excluded in the final model, thus suggesting that self-assessment of quality of life and mood changes may not be directly related to the disease progression. Therefore, our main results appear to support the hypothesis that emotional dysregulation, in this case associated with DHEA/CORT activity, could be an important factor in cancer development.

Stress can play a major role in the progression of many diseases, including cancer [49, 50]. Plenty of evidence show that DHEA and DHEA-S are involved in the individual’s response to stress and that it might provide beneficial behavioral and neuroendocrine effects, such as preventing hippocampal toxicity induced by oxidative stressors [28]. Both chemicals have been also found in the brain independently of their peripheral origin [51], and since they have been both linked to an increase in dopamine activation [52], as well as several other neurotransmitters [53], their connection with mood changes and emotional regulation is well established [54]. For example, mindfulness-based stress reduction meditation programs for cancer patients were able to increase quality of life, mood states, stress symptoms, and even increase DHEA-S levels [55]. It has been shown that DHEA can also have beneficial effects on cognition of patients undergoing chemotherapy; specifically, patients with higher DHEA-S levels before the chemotherapy had a lower probability of developing self-perceived cognitive decline [56]. Therefore, it is not surprising that patients with higher DHEA and DHEA-S in our sample also reported a higher quality of life and lower mood dysfunctions. It is also known that DHEA and DHEA-S can modulate immune functions at many levels [57]. The question remains if their protective role is confined to normal tissue, where they can even contribute to cancer progression in the context of reactive or senescent stromal microenvironment [58].

Aging is a key factor in most diseases, because many metabolic, proteomic, and immune functions become dysregulated as people growth older [59]. One of the possible mechanisms could involve a decrease in DHEA production [60]. Interestingly, the ability to release cortisol does not change significantly in aging, thus naturally decreasing the DHEA/CORT ratios and therefore increasing the probability of homeostatic imbalances in the elderly [61]. Our sample confirmed decline of DHEA and DHEA-S, but not of CORT, in urogenital cancer male patients. Age and DHEA levels were also related to the presence of metastases. Logistic regression models showed that both had an independent effect on the probability to develop metastases, but intriguingly, DHEA was identified as the most significant predictor. It would be interesting, in a future study, to test alternative mediation models to try to infer the directionality of some of the associations in the absence of possible experimental studies. This result suggests that if we can find ways to raise DHEA in older patients, there is a chance that both their quality of life and disease progression could improve. Clinical data on the effects of DHEA supplements in increasing endogenous circulating DHEA and their behavioral effects have not shown significant improvement, but one explanation is that the dosages for DHEA supplements have been too low [62]. Considering that aging is linked to a chronic oxidative and inflammatory stress, which can lead to increased inflammatory responses and accelerated cell dysfunction [63], then DHEA could play an important role in protecting patients against senescence. Further investigations are necessary to clarify how DHEA may be useful as a therapeutic tool in urogenital cancer.

The number of traumatic events experienced in the past (LEC-5) was not related to any psychological and physiological measures, which can be viewed as an indication that experiencing the negative effects of a cancer diagnosis and treatment is enough to initiate the cascade of psychobiological changes by which the external environment can influence tumor progression. The stress response can influence and support all the hallmarks of cancer that are necessary for tumor growth, including inflammation and genome instability [64], and thus our data indicate that even a single traumatic event can be the starting point for tumor growth.

Although our initial results are promising, our study had several important limitations that will need to be addressed in the future. Firstly, and considering the heterogeneity of UC patients, we need to collect more data for each individual type of cancer. With a larger sample, we would need to compare and contrast the association between resiliency and disruption of the HPA axis activity as an indicator of cancer progression in the different types of UC. This approach would allow us to investigate if the relationship between psychological and physiological emotional regulation follows a shared pattern across organs or can change dramatically depending on the affected organ. Moreover, it would be interesting to test if similar models would be valid for female patients, and thus we should include other type of cancer more common in females, such as ovarian cancer. Due to enrollment limitations, in this study, we focused on males, but considering that both psychosocial resiliency and HPA activity can have different patterns in females [65], data on how they are related to cancer progression in women is needed. Long-term studies collecting records of patients across many years and phases of the tumor development and therapy would be necessary to create a more precise framework of the changes in the relationship between tumor progression and resiliency. Also, measuring how self-evaluation of the quality of life and emotional regulation at different stages would be extremely useful in creating better models of the relationship between HPA activity, quality of life perception, and cancer. The dynamic interaction of different factors and how their relationship changes in time has been rarely study, but clearly all the mechanisms involved in the regulation of tumors are dependent on temporal trajectories.

Finally, it is important to point out once again that our results do not imply causation, due the correlational nature of the study. Therefore, we are not arguing that improving emotional regulation prior or after the diagnosis of cancer can necessarily improve survivorship and quality of life. Indeed, even if we found that emotional dysregulation does in fact impact cancer development, we do not know to what extent emotional dysregulation and its associated neurochemistry is influencing tumor progression relative to a wide range of other biological and environmental factors not assessed in the present work. In other words, although promising, our results point toward the necessity to collect more accurate data on the patients involving many aspects of the life of patients, from life events to coping skills—as well as all the necessary physiological and biological data related to the disease.

## Conclusions

Our data support the hypothesis that DHEA and DHEA-S are involved in both the perceived quality of life and mood state and in males affected by UC. It is clear that these hormones are involved in many essential biological functions related to aging, immune function, and cell growth, but it is also clear that it cannot be characterized as a “fountain of youth.” Rather, it appears to be an ideal target as a potential molecular checkpoint for the connection between cellular proliferation and macro-events involving the whole organism. Considering the pressing need to find cost-effective natural treatments for skyrocketing health costs in a progressively aging population, our results could be useful for future translational research on how improving emotional resilience can increase cancer survivorship. Further studies identifying the exact downstream mechanisms of action and possible difference in the directionality of the reciprocal relationships between coping skills and HPA axis activity are necessary. Our preliminary data indicates that investigating the psychobiological bases of cancer progression can help us better understand the mechanisms underpinning the emotional regulation processes that people adopt spontaneously to increase their chances to survive when diagnosed with urogenital cancer. The ultimate goal is to better understand how some people can benefit from a combination of life events and habits, personality traits, and physiological/genetics characteristics to extrapolate useful guidelines for the general population.
